# Co-induction of cyclooxyenase-2 and early growth response gene (*Egr-1*) in spinal cord in a clinical model of persistent inflammation and hyperalgesia

**DOI:** 10.1186/1744-8069-7-91

**Published:** 2011-11-23

**Authors:** Sharron Dolan, Peter Hastie, Claire Crossan, Andrea M Nolan

**Affiliations:** 1 Department of Biological and Biomedical Sciences, Glasgow Caledonian University, Cowcaddens Road, Glasgow, G4 0BA, UK; 2 College of Medical, Veterinary and Life Sciences, University of Glasgow, G12 8QQ, UK

**Keywords:** Inflammation, pain, hyperalgesia, Egr-1, cyclooxygenase-2, spinal cord

## Abstract

**Background:**

This study characterised the effects of persistent peripheral inflammation of the foot on pain and spinal cord expression of cyclooxygenase-1 and -2 (COX-1 and COX-2) and early growth response gene 1 (*Egr-1*), known markers of neuronal plasticity, in a clinical model of naturally-occurring inflammatory disease and hyperalgesia in sheep ('footrot'), before and after routine treatment (parenteral treatment with antibiotics and antiseptic footbathing). The temporal pattern of expression of COX-1, COX-2 and *Egr-1 *mRNA and protein were analysed using real-time PCR and Western blotting.

**Results:**

Animals affected with persistent peripheral inflammation displayed significant hyperalgesia and lameness (a proxy indicator of spontaneous pain) restricted to the inflamed limb. Hyperalgesia and lameness were significantly attenuated 1 day after treatment, and resolved further by day 7 and day 3, respectively. COX-2 but not COX-1, protein expression was up-regulated in spinal cord from lame animals on day 0, before treatment. Following treatment and attenuation of pain behaviours, levels of COX-2 returned to control levels. Significant induction of *Egr-1 *mRNA and protein were observed in spinal cord from lame animals. Three days after treatment, levels of *Egr-1 *mRNA returned to control levels, however, *Egr-1 *protein remained elevated.

**Conclusion:**

Elevated levels of spinal COX-2 and *Egr-1 *protein correlate with the presence of pain and hyperalgesia, and may underlie persistent pain, although a direct causal link has still to be established. Understanding the temporal pattern of expression of key mediators in clinical pain states may lead to better strategies to manage pain.

## Background

While experimental models of inflammatory pain have helped increase our understanding of pain mechanisms, they are sometimes limited in addressing the diverse nature of clinical pain, focussing more on short-term cellular and molecular changes. Furthermore, they do not represent the heterogeneity of clinical pain states. The present study utilized a model of naturally-occurring persistent inflammation, pain and hyperalgesia in sheep, induced by a bacterial infection of the digital tissues of the feet of ruminants, known as 'footrot' [1,2]. 'Footrot' is a painful, chronic disease of sheep, where the anaerobic bacterium *Dichelobacter nodosus *is the primary pathogen [3]. 'Footrot' induces inflammation of the digital skin and underlying tissues, and typically extends abaxially to cause separation and under-run lesions of the keratin matrix of the hoof. Footrot is considered an economically significant disease, and to have an adverse effect on animal welfare. Both body weight and wool production are adversely affected during the clinical phase of the infection [4]. Hyperalgesia has been documented in sheep with footrot [2,5] and studies have identified alterations in a number of pain-related genes in spinal cord recovered from these animals [2].

Prostaglandins (PGs), released by the action of cyclooxygenases (COX-1 and COX-2) on arachidonic acid, contribute to spinal nociception and hyperalgesia [6-9]. COX-2 is the major source of PGs in inflammatory pain, and the target for COX-2 selective non-steroidal anti-inflammatory drugs (commonly known as coxibs). COX-2 is induced in spinal cord in response to a variety of inflammatory stimuli [7,10-15], and is associated with the central component of hyperalgesia [16], and as such, may prove to be a useful marker of spinal cord plasticity underlying persistent hyperalgesia.

The transcription factor, early growth response gene 1 (*Egr-1*) also known as *zif268*, Krox-24 and NGFI-A, is also regulated by neuronal activity, and is generally considered a model system to study synaptic plasticity [17,18]. *Egr-1 *expression increases during long-term potentiation (LTP) [19,20], and is required for encoding long-lasting memories (see review by Davis et al. [18]). *Egr-1 *is rapidly induced in spinal cord in response to sensory fibre stimulation [20-22] and following peripheral inflammation [23-27], suggesting a role for *Egr-1 *regulated target gene expression and persistent cell modifications in spinal cord neuronal plasticity and persistence of pain. Evidence that *Egr-1 *induction is dependent on NMDA receptor activation [27,28], and increased intracellular Ca^2+ ^concentration (see review by Thiel et al. [29]), both triggers of activity-dependent central sensitization [30] support this hypothesis.

This study was designed to characterise the effects of long-lasting clinical inflammation on pain behaviours and spinal cord nociceptive information processing while monitoring established markers of central neuronal plasticity, COX-2 and *Egr-1*, to determine the effectiveness of treatment on resolution of these behaviours. Results show that COX-2 and *Egr-1 *mRNA and protein expression in spinal cord correlate with the presence of pain and hyperalgesia, suggesting that these mediators, and their downstream targets, contribute to pain related plasticity in spinal cord pathways.

## Results

### Resolution of hyperalgesia after treatment

**Figure 1:**
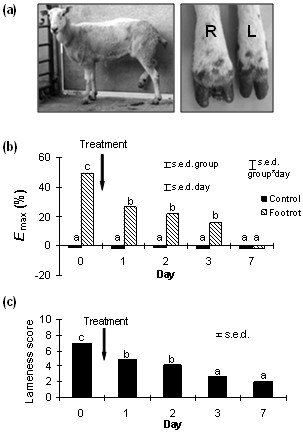
**Resolution of lameness and hyperalgesia after treatment**. Photograph of a clinically lame animal and close-up of the hindfeet (a). The animal was given a lameness score of 10 (non-weight bearing). Inflammation can be seen in the right foot and tracking up the lower limb. The magnitude of hyperalgesia (Emax%) on the lame limb on day 0 prior to treatment, and up to 7 days post-treatment is represented as the percentage change from 3 non-affected limbs according to the formula: *((mean response threshold of non-affected limbs - response threshold of lame limb)/mean response threshold of non-affected limbs) × 100*. In control animals, the mean percentage change in response threshold measured on one limb relative to the three other limbs was calculated (b). Mean lameness score for animals affected by unilateral lameness on day 0 prior to treatment for lameness, and up to 7 days post-treatment (c). Data are presented as mean values; s.e.d. represents the standard error of the difference. ^a, b, c ^Means with disparate letters are significantly different (p < 0.05).

In healthy control animals, there was no significant difference in mechanical withdrawal threshold recorded from all four legs for the duration of the study (Figure 1b). Prior to treatment, sheep affected by unilateral lameness had reduced response thresholds to mechanical stimulation in the lame limb compared to non-affected limbs and control animal limb thresholds (Figure 1b). Sheep affected by unilateral lameness displayed significant hyperalgesia (p < 0.001 vs. control sheep) on day 0 prior to treatment, which was attenuated 1 day following treatment (p < 0.001 vs. day 0), and fully resolved by day 7 after treatment (p < 0.01 vs. day 0), when thresholds were similar to those observed in control animals. Data collected from each lame sheep are presented in Table 1.

**Table 1:** Lameness, pathology and hyperalgesia score assessed in sheep before and after treatment.

**Sheep i.d**.	Lame Leg	DAY 0			DAY 3		
		***Lameness Score***	***Pathology Score***	***Emax (%) ***	***Lameness Score***	***Pathology Score***	***Emax (%)***

Y45	LH	8	4	62.6	2	4	19.2

W140	RH	8	4	47.7	1	4	25.5

G122	LF	7	4	59	3	2	-7.2

R5	RF	7	4	71.4	2	1	19.8

Y36	RH	8	3	29.3	1	1	3.7

G97	LF	6	4	62.9	4	4	62.9

B93	RF	8	2	40.7	1	0	27.9

Y56	RF	6	4	38.5	3	2	41.4

W15	LF	5	3	33.4	4	3	-13.0

237	LH	7	2	29.9	2	0	5.1

G117	RF	6	2	16.7	0	0	8.75

394	LH	4	4	25.5	5	3	3.3

B83^a^	RH	8	4	58.4	-	-	-

G60^a^	LH	6	4	32.7	-	-	-

G146^a^	RH	7	3	63.9	-	-	-

B23^a^	RH	5	2	52.7	-	-	-

W110^a^	LH	5	2	42.5	-	-	-

B80^1a^	RH	5	2	55.2	-	-	-

B1^b^	RH	6	3	47.4	3	3	-8.27

RS^b^	RH	5	4	49.5	5	3	48.9

Y192^b^	LH	5	4	52.0	4	3	31.7

B24^b^	RH	4	4	41.0	4	3	-17.5

B69^b^	LH	4	2	31.6	1	1	2.6

B93^b^	LH	4	4	37.6	2	1	2.6

***MEAN (± sem)***		**6.6 ± 0.3**		**45.1 ± 2.9**	**2.6 ± 0.3**		**15.0 ± 4.6**

*MEDIAN (range)*			**4 (2-4)**			**2.5 (0-4)**	

Mean lameness (± SEM) and median pathology (range) scores and mean magnitude of hyperalgesia (Emax), represented as the percentage change in response threshold from the non-lame limbs, for 24 sheep affected by unilateral lameness recorded on day 0 before treatment, then on day 3 after treatment. LH-left hindleg; RH-right hindleg; LF-left foreleg; RF-right foreleg; a - sheep euthanased on day 0 before treatment; b - sheep euthanased 3 days after treatment.

### Lameness Scores

On day 0 prior to treatment, mean lameness scores were 6.6 ± 0.3 in sheep affected by unilateral lameness (Table 1). There was a significant (p < 0.001 vs. day 0) decrease in lameness scores just 1 day following treatment, followed by a further significant (p < 0.001 vs. day 0) decrease in lameness 3 days post-treatment. No further resolution in lameness was observed, and some animals remained mildly lame at day 7 (1.8 ± 0.5; Figure 1c).

### Foot Pathology

On day 0 prior to treatment, the median pathology score was 4 (range 2-4); by day 3 the median score had dropped significantly (p < 0.01) to 2.5 (range 0-4), although, in 5 out of the 18 sheep examined there was no noticeable improvement in foot condition. No further improvement in foot pathology was observed by 7 days (median score 2 (range 0 - 4)).

### Lymph Node Pathology

**Figure 2:**
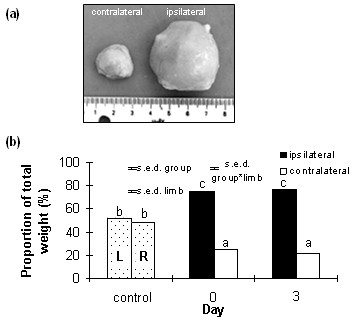
**Lymph node hyperplasia in infected sheep**. Photograph of popliteal lymph nodes ipsilateral and contralateral to inflamed limb collected from a clinically lame animal (a). Mean lymph node size (each lymph node represented as proportion (%) of combined weight) in control animals (left and right prescapular and popliteal lymph nodes) and animals affected by unilateral hindlimb (popliteal lymph nodes) and forelimb (prescapular lymph nodes) lameness on day 0 prior to treatment, and 3 days post-treatment (b). Data are presented as mean values; s.e.d. represents the standard error of the difference. ^a, b, c ^Means with disparate letters are significantly different (p < 0.001).

In control animals, left and right lymph nodes were of approximately equal size (2.0 ± 0.2 g and 1.9 ± 0.2 g, respectively; Figure 2b). In contrast, the ipsilateral lymph nodes obtained from lame animals prior to treatment (7.2 ± 0.8 g) were significantly enlarged (p < 0.001) compared to the contralateral nodes (2.9 ± 0.4 g) and compared to control sheep lymph nodes. Lymph nodes collected from animals on day 3 after treatment were still significantly enlarged (13.5 ± 2.1 g) compared to contralateral nodes (2.4 ± 0.2 g) and compared to control sheep lymph nodes.

### Expression of COX-1, COX-2 and *Egr-1 *in spinal cord

**Figure 3:**
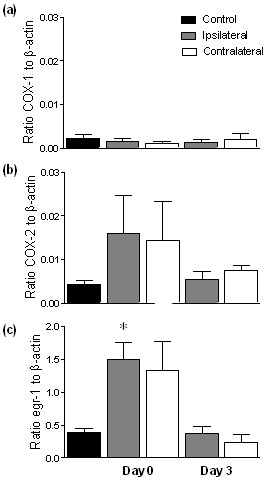
**Expression of COX-1, COX-2 and *Egr-1 *mRNA in spinal cord**. Real-time semi-quantitative measurement of COX-1 (a), COX-2 (b) and *Egr-1 *(c) mRNA in spinal cord from control sheep (n = 6) and sheep affected by unilateral lameness on day 0 (pre-treatment) (n = 6) and 3 days post-treatment (n = 6). Spinal cords were hemisected into ipsilateral and contralateral portions (data is pooled for control animals). Levels of mRNA are expressed relative to the endogenous reference gene β-actin. Significant increase from control: * p < 0.05.

COX-1, COX-2 and *Egr-1 *mRNA were constitutively expressed in spinal cord collected from control sheep (Figure 3). No change was detected in expression of COX-1 or COX-2 mRNA in ipsilateral or contralateral spinal cord in lame animals on day 0 or day 3 post-treatment compared to control animals. Significant up-regulation of *Egr-1 *mRNA was detected in ipsilateral spinal cord in lame animals on day 0 before treatment compared to control animals (4.5 fold decrease; p < 0.05). Levels had returned to control levels by day 3 after treatment.

### Expression of COX-1, COX-2 and *Egr-1 *protein in spinal cord

**Figure 4:**
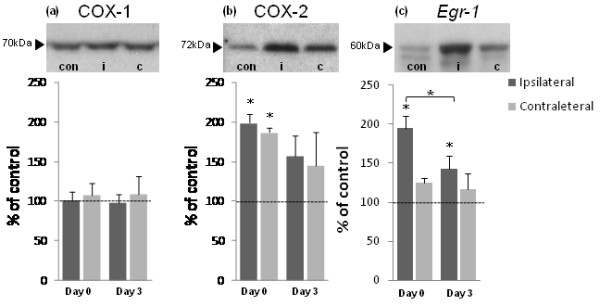
**Expression of COX-1, COX-2 and *Egr-1 *proteins in spinal cord**. Western blot analyses of COX-1 (a), COX-2 (b) and *Egr-1 *(c) protein in spinal cord. Photomicrographs show expression of COX-1, COX-2 and *Egr-1 *at 70, 72 and 60 kDa, respectively, as expected in a representative control sample (**con**) and ipsilateral (**i**) and contralateral (**c**) spinal cord from an animal affected by unilateral lameness on day 0. Graphs show densitometric quantification of levels of COX-1 (a), COX-2 (b) and *Egr-1 *(c) protein in ipsilateral and contralateral spinal cord tissues from animals affected by unilateral lameness on day 0 before treatment (n = 6) and 3 days after treatment (n = 6). Bars represent mean ± SEM protein expression (as %) relative to control levels (100%). Significant increase from control: * p < 0.05; significant decrease from day 0: * p < 0.05.

Western blot analyses of protein extracts prepared from spinal cord homogenates revealed COX-1 and COX-2 antibody labelled bands at approximately 70 and 72 kDa, respectively, as expected (Figure 4). The antibody to *Egr-1 *detected bands at approximately 60 kDa, as expected (Figure 4). A significant up-regulation in COX-2 protein expression was found in ipsilateral and contralateral spinal cord from lame animals on day 0 relative to control animals (both p < 0.05 relative to control animals (100%)). COX-2 expression returned to control levels 3 days after treatment. No change was detected in levels of COX-1 protein in spinal cord between groups. *Egr-1 *protein levels were significantly increased in ipsilateral spinal cord from lame animals on day 0 (p < 0.05 relative to control animals (100%)), and this up-regulation persisted 3 days after treatment (p < 0.05 relative to control animals (100%)). Further analyses, however, revealed that levels *Egr-1 *proteins were decreased in ipsilateral spinal cord 3 days after treatment relative to levels in lame sheep on day 0 (p < 0.05; Figure 4).

## Discussion

This study describes a unique approach to the neurobiology of the resolution of clinical inflammatory pain. The model of inflammatory pain used in this study, has been described previously and is an excellent model of naturally-occurring pain [1,2]. Strict inclusion criteria were adhered to and from clinical examination animals recruited to the study were estimated to have had the infection for more than two weeks. Therefore, this model is representative of persistent pain. The condition 'footrot' is initiated by invasion of bacteria into the interdigital tissues inducing dermatitis, and as infection spreads the bacteria penetrate further into deep tissues causing extensive tissue damage and separation of the hoof horn, accompanied by inflammation and pain [31]. To contain the clinical heterogeneity of diseased animals, only sheep with lameness scores ≥ 5 and pathology scores ≥ 2, were recruited. As expected, animals affected with unilateral inflammation displayed significant lameness (taken as a proxy indicator of spontaneous pain) and hyperalgesia, restricted to the inflamed limb. Previous studies in this laboratory revealed a significant relationship between lameness score and mechanical hyperalgesia, supporting a positive association between the magnitude of hyperalgesia and the intensity of inflammatory pain [2]. The presence of mechanical hyperalgesia localised to the inflamed limb indicates an alteration in sensory nerve function and nociceptive processing occurring centrally, as a consequence of release of pro-inflammatory mediators at the site of inflammation and at spinal cord level. Previous studies have shown that parenteral and topical antibacterial treatment produces rapid recovery (within 5 days of treatment) from foot lesions and lameness in sheep with footrot [32]. In the present study, antiseptic footbathing, trimming the hoof horn and parental antibiotics rapidly reversed both lameness and hyperalgesia, with more than 85% of animals showing significant improvements 3 days after treatment, despite the persistence of foot pathology and lymph node hyperplasia. These findings indicate that treating the primary source of infection and therefore reducing inflammation is effective and sufficient to reverse persistent pain hypersensitivity in this clinical model.

The bilateral induction of COX-2 protein in spinal cord from sheep with inflammation and subsequent decrease in expression 3 days after treatment as lameness and hyperalgesia were resolving, supports the theory derived from experimental models that that spinal COX-2 derived PGs play a key role in central mechanisms of hyperalgesia in clinical pain [33-36]. A bilateral and inter-segmental induction of spinal COX-2 in response to unilateral inflammation induced by injection of complete Freund's adjuvant (CFA) into the footpad in rat has been described previously [12,15], suggested to be due to a humoral response induced by circulating pro-inflammatory mediators such as IL-1β. A humoral immune response could account for the bilateral increase in COX-2 protein in spinal cord observed in the present study, however, no corresponding increase in levels of COX-2 were detected in spinal cord segments distal to the inflamed limb afferent termination site (data not presented). It is likely that increased noxious input from activated primary afferents from the inflamed limb, driving activity of spinal dorsal horn neurons, contribute to induction of COX-2, perhaps through activation of substance P, which is known to lead to increased expression of COX-2 and release of PGE_2 _in spinal cord [37]. Induction of COX-2 in spinal cord appears to be transient in most rodent models of inflammation, for instance, peaking 6 hours after hindpaw incision or intraplantar injection of carrageenan [38], and between 6-12 hours in response to injection of CFA into the footpad; returning to baseline levels a few days later [11,12,15], even though hyperalgesia and paw inflammation persists for several days beyond this period in this model [12,39,40]. These studies indicate that COX-2 in spinal cord plays key role in initiating central sensitization with acute inflammation. The elevated levels of COX-2 protein observed in spinal cord from sheep with persistent inflammatory disease, however, suggests that COX-2 derived PGs also contribute to maintaining the central component of hyperalgesia in this model. This is supported by a recent study by Prochazkova et al. [41] showing long-lasting up-regulation of COX-2 mRNA and protein in spinal cord in a model of osteoarthritis and persistent hyperalgesia.

Biosynthesis of *Egr-1 *mRNA and protein was increased in ipsilateral spinal cord from sheep with unilateral inflammation, also likely due to increased input from activated nociceptors in the inflamed limb. *Egr-1 *is primarily expressed in superficial dorsal horn neurons that receive input from small-diameter myelinated and unmyelinated afferent fibres [22,25], and expression is rapidly induced in these laminae in response to a variety of acute experimental inflammatory stimuli including intraplantar injection of formalin [24,26,27] and carrageenan [25], and nerve-injury [42]. Furthermore, the temporal pattern of *Egr-1 *expression is reported to parallel development of hyperalgesia and inflammation [23,25], suggesting a link between *Egr-1 *expression and behavioural responses to inflammatory pain. This hypothesis was further strengthened by a study in *Egr-1 *knockout mice by Ko et al. [43], that reported that although acute nociception is unaltered, hypersensitivity induced by formalin or CFA was diminished in these mice. A role for spinal *Egr-1 *in the maintenance of inflammatory pain is supported by evidence that *Egr-1 *antisense treatment in rat resulted in deficits in the maintenance of mechanical allodynia [21]. In the present study, the return of *Egr-1 *mRNA levels to baseline levels and decrease in protein expression 3 days after treatment, as lameness and hyperalgesia were resolving, further strengthens a role for *Egr-1 *in central neuronal plasticity underlying inflammatory or persistent pain.

*Egr-1 *activates a variety of downstream target genes by binding to the DNA sequence GCG(G/T)GGCG in the genes promoter region, including microsomal prostaglandin E synthase-1 (mPGES-1), the enzyme that couples with COX-2 to produce PGE2 [44], and COX-2 [45], suggesting an interaction between *Egr-1 *regulated transcription and inflammatory prostanoid production. This hypothesis is supported by evidence that inhibition of *Egr-1 *results in decreased COX-2 and mPGES-1 expression in LPS-stimulated murine macrophages [45], and induction of mPGES-1 mRNA following injurious ventilation in lung tissues is diminished in *Egr-1 *knockout mice [46]. Inflammatory prostaglandins also seem to play an important regulatory upstream role, acting as initiators of *Egr-1 *activity. For instance, treatment of stimulated endothelial cells with a COX-2 selective inhibitor was reported to inhibit production of *Egr-1 *mRNA and protein [47], while PGE_2 _induces *Egr-1 *mRNA expression cementoblastic OCCM periodontal cells [48]. The co-induction of COX-2 and *Egr-1 *in spinal cord from sheep with unilateral inflammation and hyperalgesia supports a link between the COX-2 signalling pathway and enhanced *Egr-1 *gene transcription, and may underlie persistent cell modifications at the spinal level and persistence of pain.

## Conclusion

These data provide valuable information on the temporal pattern of expression of plasticity-related genes in spinal cord with resolution of naturally-occurring persistent inflammatory disease and associated pain. It is hypothesised that the biosynthesis of COX-2 and *Egr-1 *contribute to pain related synaptic potentiation in spinal cord pathways by mediating long lasting changes in expression of downstream targets and persistent alterations in neuronal functioning. The rapid decrease in COX-2 and *Egr-1 *expression with diminished pain and hyperalgesia following treatment of peripheral inflammation, suggest that these mediators and their downstream targets may be targeted to prevent the evolution of persistent pain into chronic pain.

## Methods

The study was approved by the Institute's Ethics and Welfare committee and all procedures were performed according to the UK Animal Scientific Procedures Act (1986). Animals were treated in accordance with the Ethical Guidelines for Investigations of Experimental Pain in Conscious Animals as issued by the International Association for the Sudy of Pain.

### Clinical model of persistent inflammation and hyperalgesia

A total of 45 adult 'Scottish Mule' ewes were used in this study, consisting of 24 sheep affected by unilateral inflammation-induced lameness ("footrot"- Figure 1), and 21 age-matched healthy control sheep selected from the same flock for euthanasia due to poor dentition or poor fertility. All lame sheep were examined clinically by an experienced observer on day 0 (prior to treatment) and a diagnosis made as appropriate. Only animals affected by unilateral 'footrot' fitting the selection criteria (described below) were recruited into the study. Control animals were also examined to exclude the presence of any other clinical signs of disease.

All unilaterally lame sheep were scored for lameness severity using an 11-point numerical rating scale (0-10; where 0 = normal and 10 = could not be more lame, i.e. not weight bearing), as previously described [49]. In addition, the affected digit was scored for pathology using a simple 5-point descriptive scale (0-4; Table 2) as described by Ley et al. [1]. Only sheep with lameness and pathology scores of ≥ 5 and ≥ 2, respectively, were recruited onto the study. All 'footrot'-affected sheep recruited had pathological lesions indicative of duration in excess of 2 weeks.

**Table 2:** Pathology scoring system adopted to assess lesion severity in lame sheep.

Score	Criteria
0	Normal foot
1	Damage to hoof; inflamed digital tissues; no smell
2	Foot swollen and hot; inflamed digital tissues
3	Foot swollen and hot; inflamed digital tissues; broken hoof
4	Foot swollen and hot; inflamed digital tissues; broken hoof; foul odour

Following initial behavioural testing on day 0, selected animals were treated according to standard farm practice; foot paring (trimming off diseased horn), foot-bathing for 10 min in 10% zinc sulphate (Golden Hoof Plus; Sheep Fair Products Ltd., UK), and antibiotic (oxytetracycline) therapy administered parenterally (Duphacycline™ LA (Fort Dodge Animal Health Ltd., UK); 20 mg kg^-1 ^bodyweight, i.m.).

### Lymph node pathology

Left and right pre-scapular and popliteal lymph nodes were obtained following euthanasia from sheep with hindlimb lameness on day 0 (*n *= 6) before treatment, and on day 3 after treatment (*n *= 6), and weighed for size analysis to further confirm unilateral inflammation. In control sheep (*n *= 6), left and right pre-scapular and popliteal lymph nodes were collected for size comparison with those obtained from lame animals.

### Behavioural testing

Withdrawal thresholds to noxious mechanical stimulation were measured according to a method used previously by our group [50]. In lame sheep, thresholds were measured on day 0 (*n *= 24), and 1 (*n *= 18), 2 (*n *= 18), 3 (*n *= 18) and 7 (*n *= 12) days post-treatment. In control animals, thresholds were also measured on day 0 (*n *= 21), and 1, 2, 3 and 7 (*n *= 10) days post-treatment. In brief, a pneumatic device connected to a calibrated pressure meter (C9595 IS, Comark, UK) was used to push a blunt pin, positioned on the skin of the leg, with increasing force until a withdrawal response was evoked. The minimum force required to evoke a clear lifting of the limb from the ground was defined as the withdrawal response threshold. This procedure was repeated 2-4 times at each time point. Readings from the pressure meter (mBar) were subsequently converted into force (Newtons; N). A cut-off threshold of 30 N was employed.

### Real-time PCR

Real-time PCR was performed on spinal cord tissue (L6-S2) from control animals (*n *= 6) and animals affected by unilateral hindlimb lameness. Tissue was utilised from lame animals euthanased (Somulose™ (Arnolds, UK); 1 ml 10 kg^-1 ^bodyweight, i.v.) on day 0 (*n *= 6) and on day 3 (*n *= 6) post-treatment. Spinal cord samples were hemisected into ipsilateral (side of pathological lesion) and contralateral sections. Total RNA was then extracted from spinal cord using TRIZOL^® ^Reagent (Invitrogen, UK), and reverse transcribed using random hexamers (Promega, UK) and M-MLV reverse transcriptase (Invitrogen, UK). Semi-quantitative real-time PCR was performed on cDNA for ovine COX-1, COX-2 and *Egr-1 *and the endogenous reference gene β-actin (MWG-Biotech AG, Germany), using a MX3000P^® ^Q-PCR system (Stratagene, UK). The PCR reaction was performed using Thermo-Start^® ^DNA polymerase (ABgene, UK) as described in Dolan et al. [2]. Samples were run in duplicate for each quantification assay. For relative quantification of target gene mRNA the comparative C_t _(cycle threshold) method was used as described in Dolan et al. [2], which involves normalisation of the number of target gene copies to the reference genes.

### Western blotting

Western blots were performed on spinal cord (L6-S2) tissue protein extracts from control animals (*n *= 6) and animals affected by unilateral hindlimb lameness. Tissue was utilised from lame animals euthanased on day 0 (*n *= 6) prior to treatment and on day 3 (*n *= 6) post-treatment. Spinal cord samples were hemisected into ipsilateral and contralateral sections. Protein was isolated using RIPA buffer, which enables extraction of cytoplasmic, membrane and nuclear proteins, as previously described by our group [2]. Prior to loading, protein supernatants were diluted in loading buffer (NuPage^® ^LDS sample buffer, 1 × final concentration (Invitrogen, UK); 0.05 M DTT) to a final concentration of 2 mg ml^-1 ^and incubated for 10 min at 90°C. Diluted protein lysates (30 μg spinal cord) and protein molecular weight markers (SeaBlue Plus 2^® ^Pre-Stained Standard and Magic Mark™ XP (Invitrogen, UK)) were loaded onto NuPage^® ^Novex 4-12% Bis-Tris gels (Invitrogen, UK), and run for 50 min at 200 V. Proteins were transferred onto PVDF membranes (Invitrogen, UK) at 30 V for 1 h using a semi-dry blotting system. Membranes were blocked in 0.1 M PBS; 5% skimmed milk proteins; 5% BSA; 0.1% Tween^® ^20 for 1 h at room temperature. Following blocking, membranes were incubated overnight at 4°C with COX-1 (ovine polyclonal; 1:1000; Cayman Chemical, USA) or COX-2 (murine polyclonal; 1:1000; Cayman Chemical, USA) or *Egr-1 *primary antisera (rabbit polyclonal; 1:1000; Santa Cruz Biotechnology, Inc. USA) diluted in blocking buffer. Immunoblots were then washed for 3 × 5 min in 0.1 M PBS; 0.1% Tween^® ^20 prior to incubation for 1 h at room temperature in secondary antibody (donkey anti-rabbit IgG, HRP-linked whole antibody; 1:1,000; Amersham Biosciences, UK) solution diluted in blocking buffer. Immunoblots were then washed for 3 × 5 min in 0.1 M PBS; 0.1% Tween^®^20. Following washing, immunoblots were developed and visualised by enhanced chemiluminescence ECL™ detection reagents and Hyperfilm™ (Amersham Biosciences, UK). Immunoreactivity was quantified using a calibrated imaging densitometer (GS-710™, Bio-Rad UK) linked to PC-based analysis software (Quantity One^®^, Bio-Rad UK).

### Statistical analyses

The magnitude of hyperalgesia (Emax) on the lame limb is represented as the percentage change from each animal's three other non-affected limbs according to the formula: ((mean response threshold of non-affected limbs - response threshold of lame limb)/mean response threshold of non-affected limbs) × 100. In control animals, the mean percentage change in response threshold measured on one limb relative to the three other limbs was calculated. The effect of time after treatment on the magnitude of hyperalgesia or lameness was analysed as a repeated measurements design using a residual maximum likelihood (REML) program. REML analysis was carried out due to non-orthogonality as a result of the imbalance in animal numbers between groups and time. Significant differences in the magnitude of hyperalgesia or lameness were tested using least significant differences, determined using the standard error of the difference (s.e.d.) obtained from the REML analyses.

Lymph node size is represented as the percentage of the total weight that each ipsilateral and contralateral lymph node contributes according to the formula: (ipsilateral lymph node weight/(ipsilateral + contralateral lymph node weight)) × 100. Data from forelimb and hindlimb lame animals were pooled and analysed together as initial analysis indicated no difference in response between pre-scapular and popliteal lymph nodes. Lymph node data were analysed using analysis of variance, and post-hoc least significant difference tests provided tests for differences between treatment groups, and ipsilateral and contralateral lymph node.

Real-time PCR data were analysed using an out using analysis of variance adopting a general linear model routine with post-hoc Tukey's test (Mintab, v. 14). Western blot data were converted to percentages of control values and statistical significance assessed using analysis of variance (Minitab, v13.1). The 95% confidence intervals were used for comparisons between treatment groups and controls (100%).

## List of abbreviations

COX-1: cyclooxyenase-1; COX-2: cyclooxyenase-2; *Egr-1*: early growth response gene 1; LTP: long-term potentiation

## Competing interests

The authors declare that they have no competing interests.

## Authors' contributions

SD and AMN conceived and designed the study. AMN carried out the clinical assessments, while SD, PH and CC carried out nociceptive threshold tests, treatments and molecular expression studies. SD and PH carried out the statistical analysis and SD, PH and AMN were responsible for drafting the manuscript. All authors have read and approved the final manuscript.
